# Urticaria Multiforme in an Elderly Patient: A Case Report

**DOI:** 10.7759/cureus.101007

**Published:** 2026-01-07

**Authors:** Cinthya K Gutiérrez Jara, Elisa E Aparicio Sánchez, Diana V Guerrero Hernández, Valerie D Alcántara Ramírez, Salvador A Gutiérrez Ávila

**Affiliations:** 1 Dermatology, Tacuba General Hospital, Mexico City, MEX

**Keywords:** adults, arciform wheals, benign, urticaria, urticaria multiforme

## Abstract

Urticaria multiforme is a morphological variant of urticaria, clinically characterized by arciform urticarial plaques, more commonly observed in children, with a benign and self-limited course. We present the case of a 72-year-old female who acutely developed fever and a disseminated dermatosis characterized by arciform and annular urticarial plaques, erythemato-violaceous with a pale center, accompanied by angioedema of the upper lip. Given the clinical findings and in order to exclude differential diagnoses with greater systemic involvement, a skin biopsy was performed, revealing superficial dermal edema and a superficial perivascular mixed inflammatory infiltrate with few eosinophils. Based on the histopathologic evidence, which excluded major differential diagnoses such as vasculitis and erythema multiforme, as well as the absence of systemic involvement, a diagnosis of urticaria multiforme was established. Due to the extent of the lesions, systemic corticosteroid therapy was initiated, resulting in complete remission without recurrence. Finally, antibiotic use and a recent upper respiratory viral infection were identified as associated factors. This case highlights the importance of considering this diagnosis in adults, allowing for prompt recognition and avoiding overtreatment given its benign course.

## Introduction

Urticaria multiforme, considered a morphological subtype of acute urticaria, is a benign hypersensitivity skin reaction that triggers a type I immunological hypersensitivity response, resulting in the release of histamine [1]. This condition is characterized by the acute and transient appearance of skin lesions that present as erythematous, blanchable urticarial plaques with violaceous centres and annular or arched forms; it usually responds appropriately to oral antihistamines. It has traditionally been associated predominantly with the pediatric population. Consequently, its appearance in adolescents and adults is rare, posing a diagnostic challenge in these age groups. The morphological characteristics of its lesions can cause uncertainty, as they share similarities with other annular dermatoses. For example, erythema multiforme also presents target lesions, but these are fixed, with more frequent mucosal involvement and a different immunological mechanism (T-cell mediated); whereas erythema annulare centrifugum usually shows scaly edges, slower progression, and absence of intense pruritus. These clinical similarities can lead to misdiagnosis if their differences are not properly recognized [1,2]. Therefore, the aim of this presentation is to describe the case of a 72-year-old patient who presented with acute-onset urticaria multiforme associated with a viral respiratory tract infection and previous use of antimicrobials. This case highlights the importance of considering urticaria multiforme in the differential diagnosis of annular dermatoses in older adults and reinforces the need for clinical-pathological correlation for a definitive diagnosis, given the absence of findings such as fragmentation and degeneration of neutrophil nuclei, resulting in the presence of nuclear dust (leukocytoclasia) or epidermal necrosis. The favorable response to the systemic treatment implemented is also noteworthy [1,2].

## Case presentation

**Figure 1:**
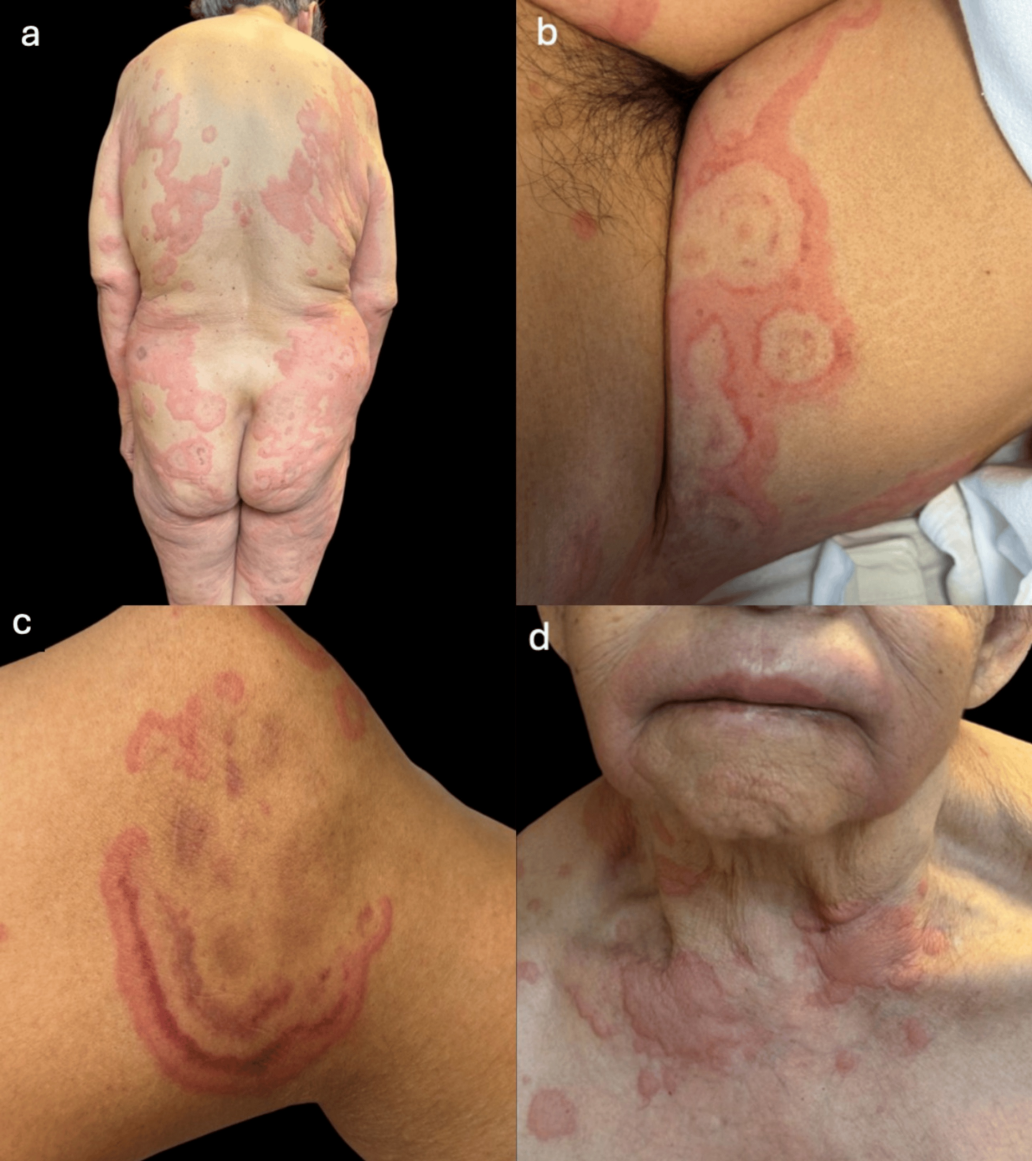
Clinical manifestations in urticaria multiforme A generalized dermatosis is observed, involving the head, neck, trunk, and both upper and lower extremities (a). It presents as multiple annular and arciform plaques with an urticarial appearance, displaying reddish-violaceous tones with pale centers (b,c), and is accompanied by angioedema (d).

A 72-year-old woman with no significant medical history presented to the emergency department with a fever of 38.7 °C and pruritic erythematous lesions. On physical examination, a disseminated dermatosis was noted on the head, anterior neck, lateral aspects of the trunk, gluteal region, and the upper and lower extremities. The findings consisted of multiple erythematous-edematous plaques, urticarial in aspect, arciform and annular in configuration, of variable size, red-violaceous with pale centers, with a smooth, elevated surface and well-defined borders, of acute onset, each lesion lasting less than 24 hours, and associated pruritus, alternating with angioedema of the upper lip (Figure 1).

Based on the clinical presentation, urticaria multiforme was initially suspected, and a skin biopsy was obtained before treatment. Because of the extensive cutaneous involvement and associated symptoms, the patient subsequently received intravenous methylprednisolone (1 g every 24 hours for three doses), followed by an oral prednisone taper (0.5 mg/kg/day with weekly dose reductions) and levocetirizine at a dose of 5 mg every 24 hours, resulting in complete remission, with no recurrence observed seven days after symptom onset.

**Figure 2:**
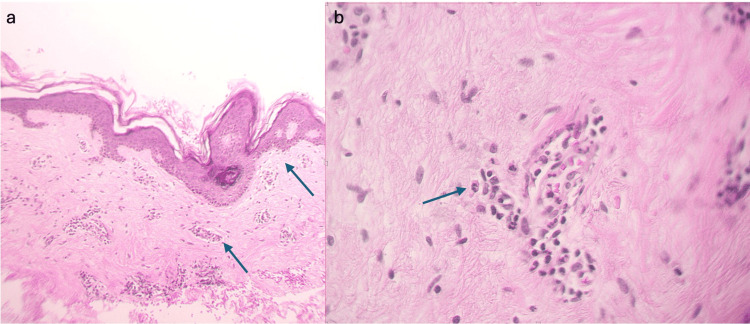
Histopathological findings The H&E-stained skin section demonstrates mild spongiosis, papillary dermal edema, and a superficial perivascular mixed inflammatory infiltrate (a), with scarce eosinophils, without evidence of vasculitis (b).

Histopathological examination showed mild spongiosis, papillary dermal edema, and a perivascular mixed inflammatory infiltrate with sparse eosinophils and absence of epidermal involvement. Notably, there was no leukocytoclasia or epidermal necrosis (Figure 2). As part of the diagnostic approach, laboratory investigations were conducted and demonstrated values within normal parameters (Table 1).

**Table 1:** Laboratory findings * abnormal values CRP - C-reactive protein; AST - aspartate aminotransferase; ALT - alanine aminotransferase; GGT - gamma-glutamyl transferase; ALP - alkaline phosphatase; TB - total bilirubin; DB - direct bilirubin; IB - indirect bilirubin

Laboratory test	Patient's value	Reference range
Hemoglobin (g/dL)	13.7	12-15.5
Hematocrit (%)	39.1	36-47
Platelets (/µL)	258,000	150,000-450,000
Leukocytes (×10³/µL)	6.2	4-11
Total neutrophils (×10³/µL)	4.2	2.2-4.8
Total eosinophils (×10³/µL)	0.03	0-0.2
Glucose (mg/dL)	110.2*	70-100
Creatinine (mg/dL)	0.62	0.6-1.3
CRP (mg/L)	3.2	<5
Urinalysis	Nitrites, glucose and protein: not detected	Not detected
AST (U/L)	18.4	10-40
ALT (U/L)	14.3	7-56
GGT (U/L)	17	9-48
ALP (U/L)	16	<20
TB (mg/dL)	0.6	0.3-1.2
DB (mg/dL)	0.3	0-0.3
IB (mg/dL)	0.3	0.2-0.9

Integration of the clinical findings with histopathologic features led to a diagnosis of urticaria multiforme. The diagnostic workup revealed a recent upper respiratory tract infection that began three days before symptom onset; however, the causative viral pathogen could not be identified because of institutional resource limitations. In addition, prior antimicrobial exposure, specifically ceftriaxone administered outside our institution at the time of onset of the respiratory symptoms, cannot be excluded as a potential triggering factor.

## Discussion

Urticaria multiforme, first described in 1937, is considered a subtype of acute urticaria and has been associated with various triggering factors, most notably infectious processes (particularly upper respiratory viral infections), medications, and vaccines. These triggers are thought to induce a type I immunologic hypersensitivity reaction, primarily mediated by IgE, leading to mast cell degranulation and histamine release [3-5]. Clinically, it manifests as transient wheals (<24 h) that coalesce into arciform plaques with violaceous centers affecting the trunk, face, and extremities. Pruritus (94%), angioedema (72%), dermographism (44%), fever (44%), and extremity edema are common features [4-6]. Although more frequent in children, anecdotal cases in adults have been reported, often associated with upper respiratory infections [7,8].

Diagnosis is primarily clinical, and the main role of skin biopsy is to exclude alternative diagnoses with potential systemic involvement, including erythema multiforme, urticarial vasculitis, serum sickness-like reactions, and acute or chronic urticaria. In this setting, differentiation between urticaria multiforme and acute urticaria relies largely on clinical features, particularly lesion morphology and associated systemic findings, rather than on pathophysiologic or histopathologic distinctions. Laboratory findings are typically normal or nonspecific, and no biochemical markers have been identified to confirm urticaria multiforme [4,8]. Distinguishing features include differences in lesion duration, the absence of histopathologic evidence of vasculitis, and the lack of recent medication exposure [5,8].

Histopathologic findings in urticaria multiforme are nonspecific and indistinguishable from other forms of urticaria. They may include dermal edema and a perivascular lymphocytic infiltrate with a few eosinophils, with emphasis on the absence of features consistent with vasculitis, such as leukocytoclasia or fibrinoid necrosis [8].

The condition is benign, with an excellent prognosis and resolution occurring approximately seven days after onset. Treatment is primarily symptomatic, with antihistamines as first-line therapy. Although international guidelines do not provide standardized dosing recommendations for the use of systemic corticosteroids in this condition, their administration in the present case was guided by the initial clinical suspicion of differential diagnoses, particularly erythema multiforme, the extensive cutaneous involvement, the significant symptom burden, the rarity of erythema multiforme in geriatric patients, and the lack of histopathological confirmation at the time treatment was initiated. In this context, systemic corticosteroids have been reported to result in favorable clinical outcomes [8-10].

Traditionally considered a pediatric condition, adult cases are uncommon and may present diagnostic challenges, particularly in older patients with comorbidities, in whom chronic illnesses and their treatments can obscure clinical interpretation [11]. This patient presented with extensive pruritic lesions accompanied by fever, prompting a broad differential diagnosis that included vasculitis, severe drug-related reactions, and autoimmune processes. In this context, histopathological evaluation was essential, as it not only supported the diagnosis of urticaria multiforme but also allowed exclusion of its principal differential diagnoses, a consideration of particular importance in adult patients, in whom alternative systemic conditions are more prevalent.

## Conclusions

Urticaria multiforme is a benign, self-limited condition that is uncommon in adults but may mimic more serious dermatoses. This case underscores the importance of considering this variant in the differential diagnosis of urticarial lesions in older adults, particularly in the context of recent exposure to potential triggers such as medications or infections. In our patient, the diagnosis was made in a geriatric individual, an age group in which this condition is distinctly uncommon, thereby necessitating a careful and systematic diagnostic approach. Initial evaluation should focus on excluding systemic involvement through appropriate laboratory testing. Although the diagnosis is primarily clinical, the atypical age at presentation and the differential considerations described above may justify histopathologic examination to exclude alternative diagnoses.

In summary, this report expands the clinical spectrum of urticaria multiforme by documenting its occurrence in a geriatric patient. Awareness of this atypical presentation may facilitate timely recognition and appropriate evaluation, thereby avoiding unnecessary investigations and interventions while ensuring exclusion of clinically relevant alternative diagnoses.
